# Patella baja after intramedullary nailing of tibial fractures, using an infrapatellar/transtendinous approach, predicts worse patient reported outcome

**DOI:** 10.1007/s00068-021-01807-9

**Published:** 2021-11-02

**Authors:** Tilman Graulich, Julius Gerhardy, Tarek Omar Pacha, Marcus Örgel, Christian Macke, Christian Krettek, Mohamed Omar, Emmanouil Liodakis

**Affiliations:** Trauma Department, Hannover Medical School, Carl-Neuberg-Straße 1, 30625 Hannover, Germany

**Keywords:** Anterior knee pain, Tibial nailing, Fracture fixation, Transpatellar approach, Patella baja

## Abstract

**Purpose:**

After intramedullary nailing of tibial shaft fractures using an infrapatellar/transtendinous approach, several patients suffer anterior knee pain. We suspect that the approach is associated with soft tissue scars and the development of a postoperative patella baja. The goal of the study is to investigate whether the development of patella baja is associated with worse subjective outcomes.

**Methods:**

We retrospectively analyzed all patients in our orthopedic trauma department between 2011 and 2020 who underwent tibial fracture fixation via intramedullary nailing via an infrapatellar/transtendinous approach. Pre- and postoperative lateral knee x-rays were evaluated by measurement of the Insall-Salvati Index, and nail tip position. All patients were asked to answer the self-assessment Kujala questionnaire and Lysholm questionnaire.

**Results:**

We included 78 patients (age: 44 ± 18 years) with a minimum follow-up of 12 months. Mean follow up was 59 ± 25 months. We included 50 male and 28 female patients. Patella baja detected by Insall-Salvati Index could be observed in 8 (10.3%) patients. Patients with patella baja showed significant worse function measured by the Kujala score 54 ± 18 vs. 80 ± 14 (*p *< 0.01). Likewise, Lysholm score did show significant differences between both groups (60 ± 24 vs. 86 ± 11; *p *< 0.01). Nail tip position was not associated with worse subjective function.

**Conclusions:**

Patella baja in patients after tibial intramedullary nailing via an infrapatellar/transtendinous approach, is associated with worse subjective function and increased pain.

## Introduction

The treatment of tibial shaft fractures with intramedullary nailing has been the golden standard in the surgical treatment of tibia fractures for decades [1, 2]. While excellent results with low complication rates are described, one of the few complications is the anterior knee pain, which is described with a heterogeneous incidence of 10–80% [3–6]. So far, most prominent reasons for anterior knee pain seems to lie in the nail entry point, the nail protrusion and the damage to soft-tissue structures such as the intrameniscal ligament, however, focusing on the nail entry point. One reason might be the iatrogenic soft tissue injury via the entry point [7–9]. Therefore, both the parapatellar- or suprapatellar entry have been described as alternative approaches. The parapatellar approach avoids the injury of the patellar tendon, however, an increased retropatellar contact force is described, which might lead to an increased osteochondral injury [4]. In a review article by Goa et al. no differences in a supra- or infrapatellar approach could be stated [6]. The stated retropatellar scaring might result in a reduced clinical function and elevated pain levels [10]. From patients after total knee arthroplasty, we know that the relation of joint line to patella height is one major factor influencing postoperative function and patient’s satisfaction. Whereas, on the one hand, a patella pseudo baja, with a raised joint line and relatively lowered patella, is known to negatively influence postoperative function, the influence of a true patella baja (PB) due to scarring of the patellar tendon and its retinacula has so far remained of minor importance [11]. However, we know from various studies on knee arthroplasty that a not inconsiderable part of anterior knee pain is caused, by access morbidity and the development of a PB [11]. Focusing back on patients with intramedullary nailing at the knee, only limited data exist reporting a development of PB in a case of retrograde nailing of the femur and one study after IM nailing of the tibia [10, 12]. Although the second study, including 33 patients after tibial nailing, observed a development of postoperative PB compared to the healthy side, no difference in anterior knee pain could be observed [12].

The aim of this study is therefore to determine the effect of intramedullary nailing of proximal (type 41-A2 and -A3 according to AO) and diaphyseal tibial fractures (type 42-A to -C according to AO) via an infrapatellar/transtendinous approach, on the sagittal patellofemoral alignment, to evaluate the knee joint function.

## Materials and methods

### Patients collective

**Fig. 1:**
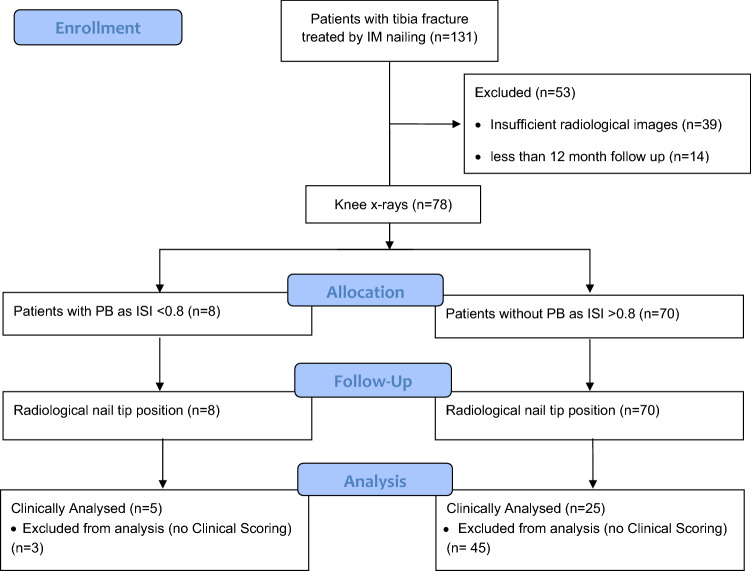
Study collective

All patients in our trauma department who suffered a tibial fracture type 41-A2 and -A3 and type 42-A till -C according to AO-classification and were treated with an intramedullary nail between 2011 and 2020 were included. Patient with a follow up of less than 12 months were excluded. All patients were treated with an Expert Tiba nail^®^ (Synthes GmbH, Oberdorf, Switzerland) (Fig. 1).

### Operative strategy

Patients were placed in supine position under general or local anesthesia. An approximately 3–5 cm long longitudinal skin incision was performed at the flexed knee above the patellar tendon and soft tissue was retracted medially and laterally. A 3–4 cm long incision of the patella tendon sheet was performed, the tendon was split longitudinally and the nail entry point was found under a.p. and lateral radiological image intensifier control with a curved awl. The nail entry point was opened, a guide wire was inserted and the intramedullary channel was reamed if applicable. Reduction was achieved using the nail and via the help of poller screws if needed [13]. Control of length, torsion, and mechanical axis was performed using clinical and radiological methods such as the cable method and the cortical step sign [14–16]. Depending on fracture morphology the nail was either locked dynamically of statically. The wound was excessively rinsed with water and wound closure was performed in layers with closure of the patella tendon sheath, subcutaneous tissue and skin.

### Radiological measurements

**Fig. 2:**
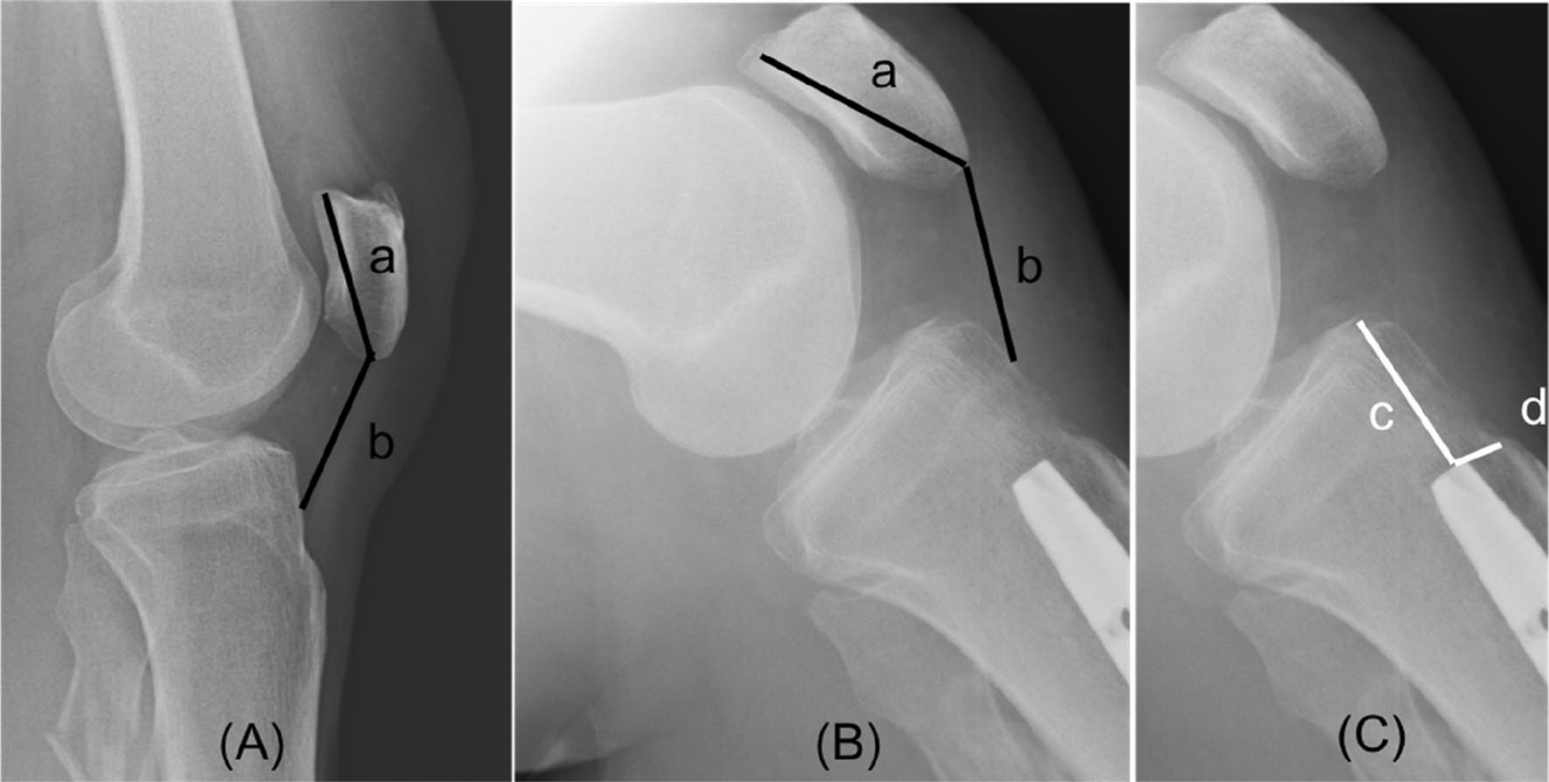
**A** Preoperative measurement of ISI by dividing of (**b**) distance of patellar tendon length and **a** patella length, B: Postoperative measurement of ISI by dividing of (**b**) distance of patellar tendon length and **a** patella length, **C** Determining of nail tip position by measurement of the distances: **c** distance nail tip to tibial tubercle, **d** distance nail tip to articular surface

Pre- and postoperative lateral x-rays were obtained, if possible, in 30° flexed knees in ap- and lateral view. An exact lateral radiograph was defined by the projection of both posterior femoral condyles above each other. Images were analyzed by a trained orthopedic surgeon with the PACS (Centricity Enterprise Web version 3.0, GE Medical Systems, Milwaukee, WI, USA). Radiological measurements included for detection of PB the measurement of Insall-Salvati Index (ISI). The distance of the patellar articular surface and the distance from the inferior patellar articular surface to the tibial tubercle was measured. By dividing both distances the ISI was determined. The presence of PB was defined as ISI < 0.8 [9, 10, 12, 13]. Nail tip position was measured. Therefore, the distance from nail tip to tibial plateau and from nail tip to tibial tubercle was measured (Fig. 2).

### Questionnaire

During outpatients visit the self-assessment questioners Lysholm score and Kujala score were answered by the patients. Regular follow up was performed at 6 and 12 weeks and at 6 and 12 months. Further follow up was performed every year. Due to the retrospective nature of this study, patients who were not within the regular follow up or did not want to participate personally were given a three-time phone call to answer questions. If this was not successful questionnaires were sent via post. All, fully completed questionnaires were included. The Lysholm score has a range from 0 to 100 points, with high points indicating good function and low points indication poor function. This is a well-established score in patellofemoral pain and patellofemoral instability [21]. The score was established in a collective of 488 people with an average score of 94 (range 43–100) [22]. Excellent results are categorized as (95–100), good results are categorized as (84–94), fair results are categorized as (65–83), and poor results are categorized as (≤ 64). The validity is reported to be convergent to the Kujala scores [23]. However, the Kujala score is focused and validated specifically for anterior knee pain. Likewise to the Lysholm score, the Kujale score ranges from 0 to 100 with high scores indicating good outcome.

### Institutional Review Board (IRB)

Informed consent was obtained from all individual participants who were included in the study. The study was approved by the local ethics review board (No. 9160_BO_K_2020).

### Statistical analysis

As the trial was of exploratory nature, no sample size calculation was performed. Data were summarized using Microsoft Excel^®^ software and statistical analysis was performed with IBM SPSS Statistics^®^ Version 25. For categorical values, the Fischer’s exact test and the Pearson-Chi-square test were used. Continuous data were tested for normal distribution with the Kolmogorov Smirnoff Test. If data were normally distributed an independent samples *t* test was used. If data were not normally distributed the non-parametric Mann–Whitney *U* test was used. A post how power analysis was performed using G-Power (Version 3.1). Data were described by mean ± standard deviation defining significance level at *α* = 0.05.

## Results

### Demographic data

Out of 131 tibial fractures we included 78 tibial fractures, which were classified as type 41-A2 or -A3 or type 42-A till -C according to AO-classification. Follow up included evaluation of clinical and radiological function. Out of these 131 fractures, 39 tibial fractures were excluded due to insufficient lateral knee imaging. Out of the remaining 92 tibial fractures, further 14 patients were excluded due to a follow up of less than 12 months, leaving 78 patients for final inclusion. We included 50 male and 28 female patients with a mean age of 44 ± 18 years. Mean follow up was 59 ± 25 months. There was no difference between patients with PB and without PB regarding, sex, age and time of follow up (Table 1).

**Table 1:** General patients’ data

	Total	PB (*n* = 8)	No PB (*n* = 70)	*p* value
Male/female	50/28	5/3	45/25	0.922
Age (years)	44.50 ± 18.31	44.89 ± 21.68	44.29 ± 18.75	0.933
FUP (month)	59.14 ± 25.57	59.67 ± 29.55	58.82 ± 23.15	0.924
Operation time (minutes)	199 ± 122	275 ± 134	195 ± 109	0.060
Complication	14	1	13	0.676

### Radiological measurements

On lateral knee x-ray images, the ISI was determined. The mean postoperative ISI was 1.01 ± 0.19 compared to preoperative ISI with 1.00 ± 0.19 (*p *= 0.682). A high correlation of 0.7 was observed between pre- and postoperative ISI (*p *< 0.01). Postoperatively, PB was observed in 8 (10.3%) patients with an ISI of 0.66 ± 0.09 compared to 70 (89.7%) patients without PB and an ISI of 1.05 ± 0.15 (*p *< 0.01). Distance from nail tip to tibial tubercle and from nail tip to articular surface did not show any differences between both groups. There was no difference in pre- and postoperative ISI. Therefore, preoperative ISI was no indicator for postoperative ISI (Table 2).

**Table 2:** Clinical and radiological outcome scores

	Total	PB	No PB	*p* value
ISI	1.01 ± 0.19	0.66 ± 0.09	1.05 ± 0.15	**0.00001**
NS	15.04 ± 8.63	13.25 ± 8.19	15.06 ± 8.82	0.583
NT	16.07 ± 6.83	14.33 ± 6.59	16.12 ± 7.21	0.506
Lysholm score	84.02 ± 16.35	60.60 ± 24.76	86.36 ± 11.77	**0.001**
Kujala score	78.40 ± 17.98	54.00 ± 18.88	80.24 ± 14.81	**0.002**

A *p*-values ≤ 0.05 was considered as statistically significant

*ISI* Insall Salvati Index, *NS* Distance Nail tip to articular surface, *NT* Distance Nail tip to tibial tubercle

### Patient reported outcome measurements

The overall Lysholm score was 84 ± 16 points which indicated as a fair result. In patients with PB the Lysholm score was 60 ± 24 which was categorized as poor, whereas patients without PB had a Lysholm score of 86 ± 11 which was categorized as good (*p *= 0.001). The overall Kujala score was 78 ± 17 points. We observed significant worse patient reported outcome scores in patients with PB (54 ± 18 points) compared to those without PB (80 ± 24 points). (*p *= 0.002). In both, Kujala- and Lysholm score, questions regarding knee pain level showed significant worse scores (*p *= 0.027 and *p *= 0.002) (Table 3).

**Table 3:** Kujala- and Lysholm score questions

	Question	Total	PB (*n *= 5)	No PB (*n *= 25)	*p* value
K1	Limp	3.73 ± 1.43	2.80 ± 1.78	3.88 ± 1.01	0.067
K2	Support	4.71 ± 0.71	3.80 ± 1.09	4.75 ± 0.67	**0.016**
K3	Walking	4.26 ± 1.26	2.80 ± 2.16	4.60 ± 0.82	**0.003**
K4	Stairs	8.87 ± 1.81	7.00 ± 2.44	9.08 ± 1.71	**0.041**
K5	Squatting	4.17 ± 1.77	2.80 ± 1.92	4.24 ± 1.30	**0.046**
K6	Running	4.73 ± 4.54	0.60 ± 1.34	4.87 ± 4.50	**0.048**
K7	Jumping	5.48 ± 4.24	2.80 ± 4.17	5.12 ± 4.27	0.276
K8	Prolonged sitting with knee flexed	8.09 ± 3.18	5.20 ± 2.68	8.48 ± 3.07	**0.035**
K9	Pain	8.61 ± 2.49	5.80 ± 4.14	8.84 ± 2.32	**0.027**
K10	Swelling	9.14 ± 1.47	8.80 ± 1.78	9.12 ± 1.53	0.681
K11	Abnormal painful kneecap movement	9.19 ± 2.02	7.60 ± 3.28	9.36 ± 1.80	0.095
K12	Atrophy of thigh	4.16 ± 1.68	2.60 ± 2.51	4.36 ± 1.47	**0.039**
K13	Flexion deficiency	3.88 ± 1.68	2.80 ± 1.78	4.12 ± 1.50	0.094
L1	Limp	3.80 ± 1.31	3.00 ± 0.00	3.96 ± 1.01	**0.047**
L2	Using cane or crutches	4.90 ± 0.43	4.20 ± 1.09	5.00 ± 0.00	**0.000**
L3	Locking sensation in the knee	13.45 ± 3.26	15.00 ± 0.00	13.32 ± 3.21	0.258
L4	Giving way sensation from knee	20.71 ± 6.30	12.00 ± 10.36	21.60 ± 4.72	**0.002**
L5	Pain	20.95 ± 6.55	11.00 ± 10.83	21.80 ± 5.18	**0.002**
L6	Swelling	8.52 ± 2.61	7.60 ± 3.58	8.32 ± 2.81	0.620
L7	Climbing stairs	8.28 ± 2.52	6.00 ± 2.82	8.72 ± 2.23	**0.024**
L8	Squatting	3.38 ± 1.84	1.80 ± 2.05	3.64 ± 1.65	**0.037**

A *p*-values ≤ 0.05 was considered as statistically significant

## Discussion

In our retrospective analysis of a single canter study in a cohort of 78 patients, we had two major observations. First, we could observe PB with an incidence of 10.3%. And secondly, those patients with a postoperative PB showed a significant worse outcome in patellofemoral specific functional score. Preoperative ISI did not predict worse postoperative ISI or worse postoperative subjective knee function.

Although, intramedullary nailing is a well-established and successful treatment option for patients with tibial shaft fractures a high complication rate of 10–80% of the patients who suffer anterior knee pain is described [3–6]. Several reasons for anterior knee pain have been described and one major reason is the iatrogenic soft tissue damage with potential painful scarring resulting in a shortening of the patellar tendon [12]. Turkmen et al. could show that a shorter patellar tendon can be observed in patients after intramedullary nailing on the operated side, compared to the non-operated side [12]. Contradictory to Turkmen et al. we did not observe a general postoperative shortening of the patellar tendon compared to preoperative x-ray images [12]. One reason might be the inherent inaccuracy of the radiological imaging. For reproducible lateral x-ray images, a 30° knee flexion is needed. However, due to initial posttraumatic pain at the fracture side this might not have been possible in any case. Furthermore, compared to Turkmen et al. we did observe worse subjective function and knee pain in patients with patella baja [12]. One reason might be the measured score. Both scores, the Lysholm score and the Kujala score are validated convergent scores. Although a minimum clinically important difference (MCID) has not been determined so far, both scores are validated for knee pain [21]. However, most importantly, out of 100 points in the Lysholm score, 60 points are not specifically focused on pain and instability in the patellofemoral joint, making this score a valuable interpreter of general knee function but less for interpretation of specific anterior knee pain [24]. Likewise to the Lysholm score, the German version of the Kujala score was validated, too [23]. However, it is focused and validated specifically for anterior knee pain. Likewise, to the Lysholm score the Kujala score ranges from 0 to 100 with high scores indicating good outcome. Whereas in healthy controls an average score of 99.9 is reported, patients with anterior knee pain report an average score of 82.8 and patients with patella instability report an average score of 62.2. Interestingly, the general Kujala score in our collective, with 78 ± 17 points, was even worse than in in the stated literature indicating a general higher degree of anterior knee pain in our collective. For interpretation of differences between the groups the minimal detectable change (MDC) was 13 points (CI = 95%) for the Kujala score. Therefore a change of at least 13 is needed to reflect a true change between the groups [25]. Therefore, our observed difference of 16 points between patients with PB (54 ± 18) and patients without PB (80 ± 14) is not only statistically significant but also a detectable clinical difference between both groups. Comparing the Lysholm score between both groups significant worse scores were observed in patients with PB and patients without PB. Interestingly both scores showed significant worse scores concerning questions on knee pain.

Another reason which has to be discussed is the potential iatrogenic injury via the entry point [7, 8]. In an eight year long term follow up of an randomized controlled trail comparing transpatellar- and parapatellar approach no differences in the rate of anterior knee pain could be observed [26]. However, the parapatellar approach results in an increased retropatellar contact force which might lead to an increased osteochondral injury via the parapatellar approach [4]. Furthermore, in a review article by Goa et al. no differences in a supra- or infrapatellar approach could be stated [6]. However, the authors state that no longtime follow up exists on the longtime results of possible retropatellar injuries. While a general trend towards a better outcome for a suprapatellar than for the infrapatellar/transtendinous entry point for the nail is described in a meta-analysis comparing the two methods, any iatrogenic retropatellar cartilage damage is yet ignored, so that the infrapatellar/transtendinous approach is still used most often [6].

We could show, that a significant association of postoperative development of PB, is associated with a worse Kujala- and Lysholm score and a higher general pain level. Therefore, we believe that to a certain degree the development of PB after tibial nailing via infrapatellar/transtendinous entry point should be avoided. As one therapeutic option we would suggest careful reconstruction of anatomical structures with suture of patella tendon sheet and subcutaneous skin closure. Weather this might reduce the rate of PB and reduce the rate of anterior knee pain needs to be evaluated in further studies.

There are several limitations to this study. First, the high rate of patients with no clinical scoring (62%). Second, preoperative lateral knee x-ray are not always performed in 30° flexed knees, decreasing the accuracy of the radiological measurement. This study has also important strengths. To the best of our knowledge this is the first study to evaluate pre- and postoperative patella baja showing, that the preoperative patellar position is no individual indicator for worse postoperative knee function. Moreover, we did a post hoc power analysis showing a high power of 90.6% for Kujala score and even more for Lysholm score with 97%.

## Conclusion

Patella baja in patients after tibial intramedullary nailing via an infrapatellar/transtendinous approach can be observed in more than 10% and is associated with worse subjective function and increased pain.

## Data Availability

Not applicable.
